# N-acetylcysteine alleviates post-resuscitation myocardial dysfunction and improves survival outcomes via partly inhibiting NLRP3 inflammasome induced-pyroptosis

**DOI:** 10.1186/s12950-020-00255-3

**Published:** 2020-08-05

**Authors:** Fenglian He, Guanghui Zheng, Jingying Hou, Qiaohua Hu, Qin Ling, Gongfa Wu, Hui Zhao, Jin Yang, Yue Wang, Longyuan Jiang, Wanchun Tang, Zhengfei Yang

**Affiliations:** 1 The Second Hospital of Anhui Medical University, Hefei, 230032 China; 2 Sun Yat-sen Memorial Hospital, Sun Yat-sen University, 107 Yan Jiang Xi Road, Guangzhou, 510120 China; 3 Weil Institute of Emergency and Critical Care Research, School of Medicine, Virginia Commonwealth University, Richmond, VA 23284 USA; 4 Zeng Cheng District People’s Hospital of Guang Zhou, Guangzhou, 511300 China

**Keywords:** N-acetylcysteine, Cardiopulmonary resuscitation, Myocardial function, Pyroptosis

## Abstract

**Background:**

NOD-like receptor 3 (NLRP3) inflammasome is necessary to initiate acute sterile inflammation. Increasing evidence indicates the activation of NLRP3 inflammasome induced pyroptosis is closely related to reactive oxygen species (ROS) in the sterile inflammatory response triggered by ischemia/reperfusion (I/R) injury. N-acetylcysteine (NAC) is an antioxidant and plays a protective role in local myocardial I/R injury, while its effect on post-resuscitation myocardial dysfunction, as well as its mechanisms, remain elusive. In this study, we aimed to investigate the effect of NAC on post-resuscitation myocardial dysfunction in a cardiac arrest rat model, and whether its underlying mechanism may be linked to ROS and NLRP3 inflammasome-induced pyroptosis.

**Methods:**

The rats were randomized into three groups: (1) sham group, (2) cardiopulmonary resuscitation (CPR) group, and (3) CPR + NAC group. CPR group and CPR + NAC group went through the induction of ventricular fibrillation (VF) and resuscitation. After return of spontaneous circulation (ROSC), rats in the CPR and CPR + NAC groups were again randomly divided into two subgroups, ROSC 6 h and ROSC 72 h, for further analysis. Hemodynamic measurements and myocardial function were measured by echocardiography, and western blot was used to detect the expression of proteins.

**Results:**

Results showed that after treatment with NAC, there was significantly better myocardial function and survival duration; protein expression levels of NLRP3, adaptor apoptosis-associated speck-like protein (ASC), Cleaved-Caspase-1 and gasdermin D (GSDMD) in myocardial tissues were significantly decreased; and inflammatory cytokines levels were reduced. The marker of oxidative stress malondialdehyde (MDA) decreased and superoxide dismutase (SOD) increased with NAC treatment.

**Conclusions:**

NAC improved myocardial dysfunction and prolonged animal survival duration in a rat model of cardiac arrest. Moreover, possibly by partly inhibiting ROS-mediated NLRP3 inflammasome-induced pryoptosis.

## Background

The prognosis of cardiac arrest remains poor even after ROSC [1]. Myocardial dysfunction is one central cause of post-resuscitation circulatory failure after ROSC, which is associated with global myocardial I/R injury. During the reperfusion period, especially within the first minutes, a surge of ROS is released as a result of cardiac injury [2]. Furthermore, occurring minutes to hours after reperfusion, a secondary inflammatory response injury takes place when I/R injury and ROS induce the activation of NLRP3 inflammasome [3–6].

NAC is a sulfur-containing amino acid, which has a variety of properties, including antioxidant and anti-inflammatory qualities. Studies suggest that a supply of NAC has a therapeutic effect in various diseases [7]. In local myocardial I/R injury, NAC remarkably attenuates oxidative stress and inflammation by inhibiting the release of inflammatory mediators [8, 9]. ROS, which is released during the I/R process, promotes tissue inflammation, and activates immune responses through NLRP3 inflammasome, inducing the secretion of pro-inflammatory cytokine Interleukin 1β(IL-1β) and cell pyroptosis [10]. Recent studies showed that NAC suppressed cardiomyocytes pyroptosis by inhibiting ROS in a myocardial infarction model [11–13].

Pyroptosis is a lytic type of cell death, the process of which includes nuclear condensation, DNA damage, cell swelling, and finally, cell lysis [14, 15]. It is associated with NLRP3 inflammasome, including NLRP3, ASC, and pro-caspase-1 [16], and is inherently related to a protein named GSDMD. GSDMD is required for pyroptosis induction after NLRP3 inflammasome activation and is processed by caspase-1, but not by apoptotic caspases [17]. Pyroptosis, as an innate immune effector mechanism, emerges in various cell types [18, 19], and can be triggered by microbial infection and other stimuli [20, 21]. Moreover, recent researches have demonstrated that I/R injury induces NLRP3-dependent caspase-1 activation and triggers pyroptosis [16, 22]. However, NLRP3 inflammasome-induced pyroptosis in global myocardial I/R injury is still unclear.

In this study, we investigated the effect of NAC on post-resuscitation myocardial dysfunction and survival outcomes in a rat model of cardiac arrest, and its possible underlying mechanism. We hypothesized that NAC attenuates myocardial dysfunction and improves survival outcomes after resuscitation by partly inhibiting ROS-mediated NLRP3 inflammasome-induced pyroptosis.

## Results

### Baseline physiological parameters and CPR characteristics

In this study, a total of 65 rats were utilized, including 12 rats in the sham group.

Five rats were excluded during the experiment, of which two were due to a malfunction of ventilation, while the remaining three rats failed to successfully resuscitate.

There were no obvious differences (*P* > 0.05) in baseline physiological parameters and myocardial function among the three groups. No significant differences (*P* > 0.05) in CPR characteristics were observed between the CPR and CPR + NAC groups (Table. 1).

**Table 1:** Baseline physiological parameters and CPR characteristics in groups

Variables	Sham (*n* = 12)	CPR (*n* = 24)	CPR + NAC (*n* = 24)
Body weight (g)	517 ± 22	514 ± 21	521 ± 15
Blood temperature (°C)	36.9 ± 0.3	36.8 ± 0.2	36.7 ± 0.3
Heart rate (beats/min)	411 ± 27	410 ± 29	409 ± 26
ETCO_2_ (mm Hg)	42 ± 3	42 ± 2	42 ± 3
MAP (mm Hg)	120 ± 7	119 ± 10	120 ± 6
Arterial PH	7.40 ± 0.04	7.40 ± 0.03	7.40 ± 0.04
EF (%)	71	74	73
CO (ml/min)	114	115	112
MPI	0.604	0.602	0.611
Rate of ROSC	–	20/24	21/24
CPP in PC1 (mm Hg)	–	25.1 ± 1.2	25.4 ± 1.1
CPP in PC3 (mm Hg)	–	24.9 ± 1.3	24.2 ± 1.7
CPP in PC7 (mm Hg)	–	32.2 ± 1.8	32.1 ± 2.1

*CPR* Cardiopulmonary Resuscitation, *NAC* N-Acetylcysteine, *ETCO*_*2*_ End-Tidal CO_2_, *MAP* Mean Arterial Pressure, *PH* Potential of Hydrogen, *EF* Ejection Fraction, *CO* Cardiac Output, *MPI* Myocardial Performance Index, *ROSC* Restoration of Spontaneous Circulation, *CPP* Coronary Perfusion Pressure, *PCn* n minute after Precordial Compression,

### Post-resuscitation myocardial dysfunction and survival outcomes after ROSC

**Fig. 1:**
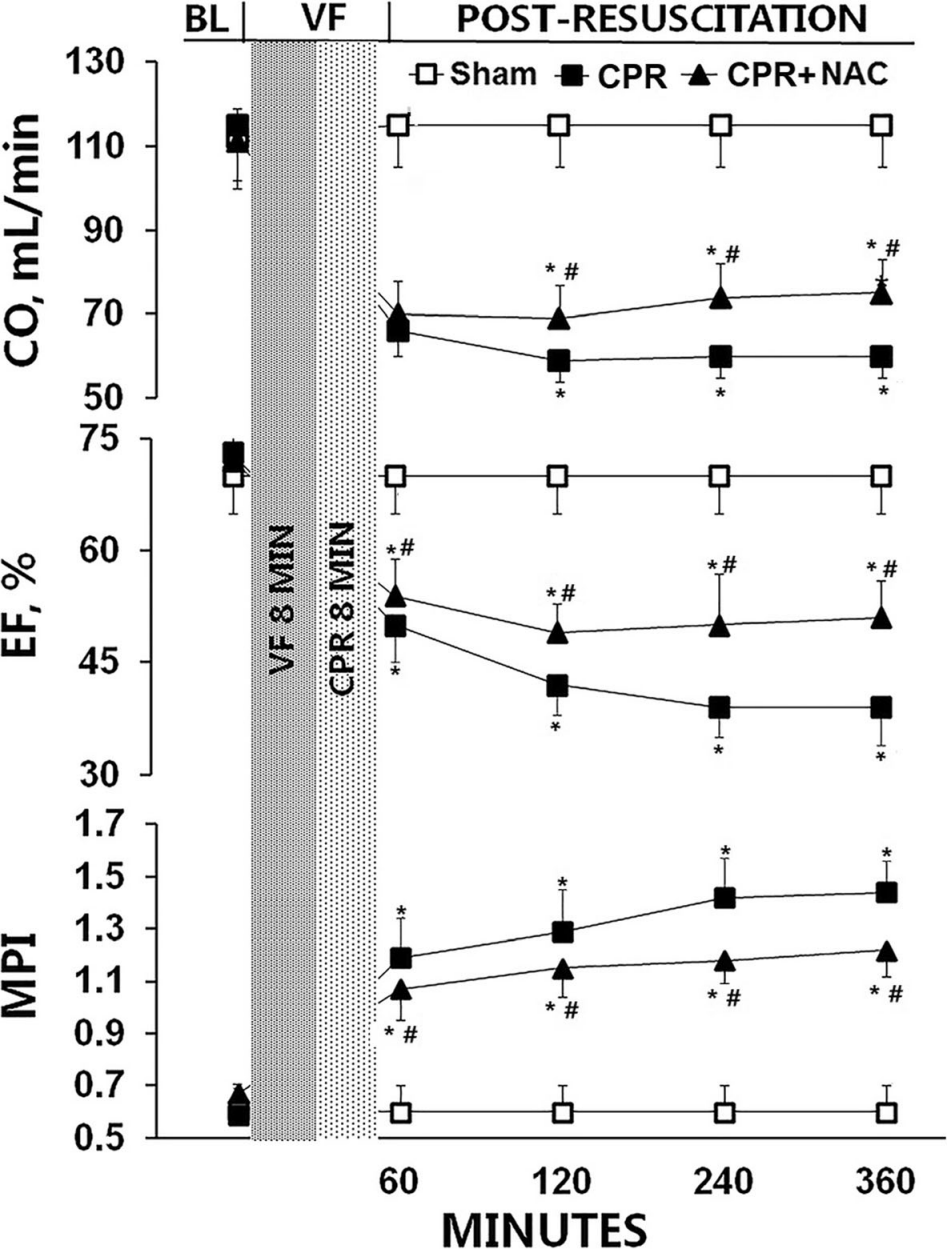
The post-resuscitation myocardial dysfunction. BL, baseline; VF, ventricular fibrillation; CO, cardiac output; EF, ejection fraction; MPI, myocardial performance index; CPR, cardiopulmonary resuscitation; CPR group, *n* = 16; CPR + NAC treatment group, *n* = 16; Sham group, *n* = 12;* *P* < .01 vs. the Sham group, ^#^*P* < .01 vs. the CPR group

**Fig. 2:**
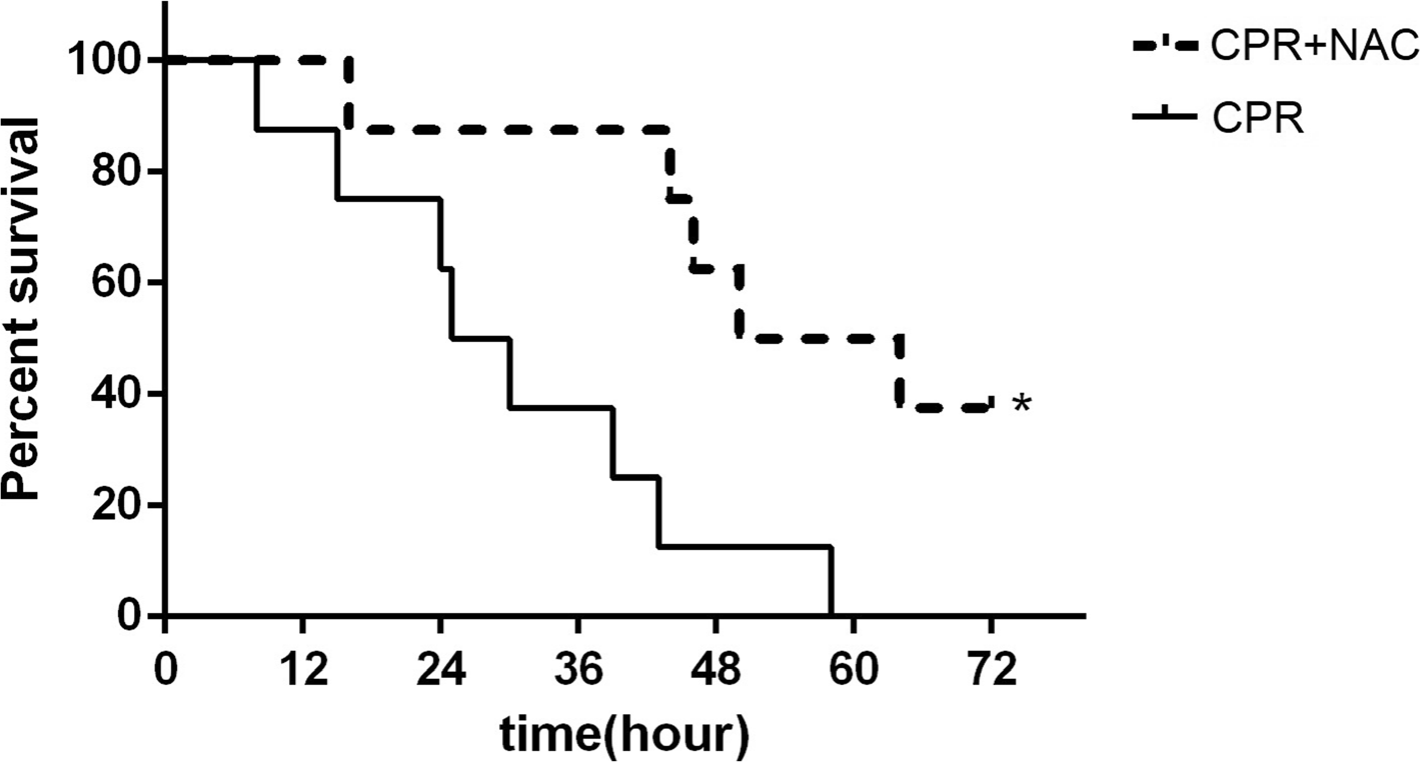
Survival outcome after ROSC. Kaplan-Meier survival curves among CPR + NAC and CPR subgroups. Log rank test displayed for 72 h survival; NAC significantly prolonged the animal survival time. ROSC, return of spontaneous circulation; CPR, cardiopulmonary resuscitation; NAC, N-Acetylcysteine. CPR + NAC treatment group, *dashed line*; CPR + NAC group, *n* = 8, *Solid line*; CPR group, *n* = 8. *P < .05 vs. the CPR group

After resuscitation, a decrease in ejection fraction (EF) and cardiac output (CO), and an increase in myocardial performance index (MPI) were observed in all the animals. After administration of NAC, EF and CO in the heart were significantly improved, especially at 6 h after ROSC when compared with the CPR group (EF and CO at 6 h increase by 22.1 and 18.3%, respectively, *P* < 0.01, Fig. 1). MPI in the CPR + NAC group exhibited a clear reduction when compared with the CPR group (MPI at 6 h decrease by 17.9%, *P* < 0.01, Fig. 1). Kaplan-Meier survival analysis showed that treatment with NAC significantly increased the survival duration, the median survival time was 54.64 ± 19.59 h while it was 36.75 ± 15.28 h in the CPR group (*P* < 0.05, Fig. 2). The cause of death was diagnosed depending on symptoms, observable signs from the animal and autopsy, and the main causes were myocardial and neurological dysfunction.

### Expression of inflammatory cytokines and oxidative stress after ROSC

**Fig. 3:**
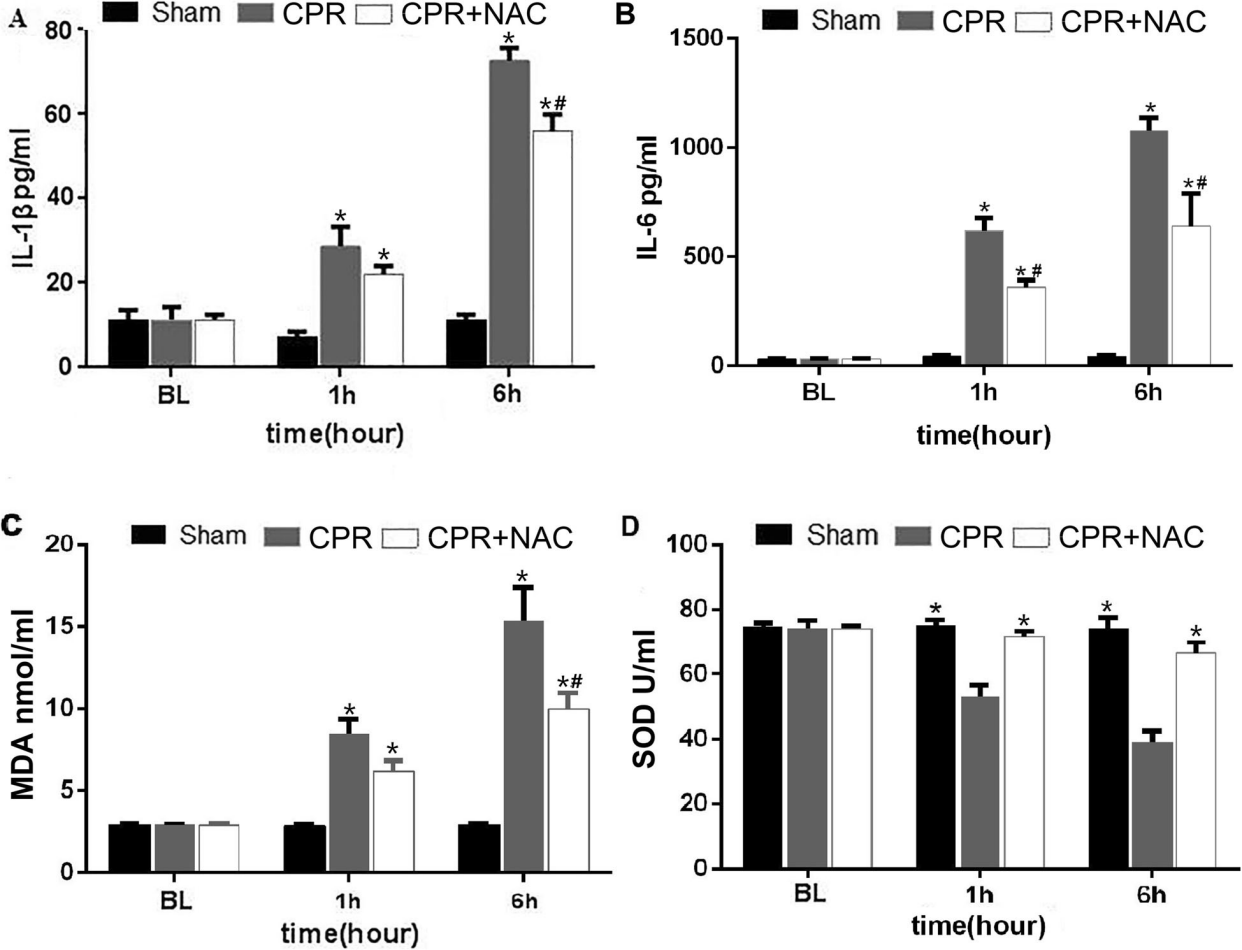
Levels of inflammatory cytokines (IL-1β and IL-6), SOD, and MDA in serum at the different groups, and the effects of NAC on these inflammatory cytokines and oxidative stress-related indices. **a** IL-1β, **b** IL-6, **c** SOD, **d** MDA. The columns represent means ± SD of three independent experiments. NAC, N-Acetylcysteine; CPR group, *n* = 16; CPR + NAC treatment group, *n* = 16; Sham group, *n* = 12. * *P* < 0.01 vs. Sham group; # *p* < 0.01 vs. the CPR group

The levels of inflammatory cytokines IL-1β and Interleukin 6 (IL-6) in serum were increased at 1 h and 6 h, especially at 6 h after ROSC in the CPR and CPR + NAC groups, compared with the sham group; both IL-1β and IL-6 levels were lower in the CPR + NAC group compared with the CPR group (IL-1β and IL-6 at 6 h increase by 22.6 and 38.55%, respectively, *P* < 0.01, Fig. 3a, b). Overall a reduction in the inflammatory response was observed after treatment with NAC. The levels of SOD and MDA were also examined in serum at baseline, 1 h and 6 h after ROSC. SOD decreased and MDA increased at 1 h and 6 h, especially at 6 h after ROSC when compared with the baseline, but SOD increased and MDA was mitigated with NAC treatment compared with the CPR group(SOD at 6 h increase by 39.9%, and MDA at 6 h decrease by 26.4%, respectively, *P* < 0.01, Fig. 3c, d). Results showed that NAC significantly reduced the levels of MDA and increased SOD.

### Expression of NLRP3 inflammasome and GSDMD after ROSC

**Fig. 4:**
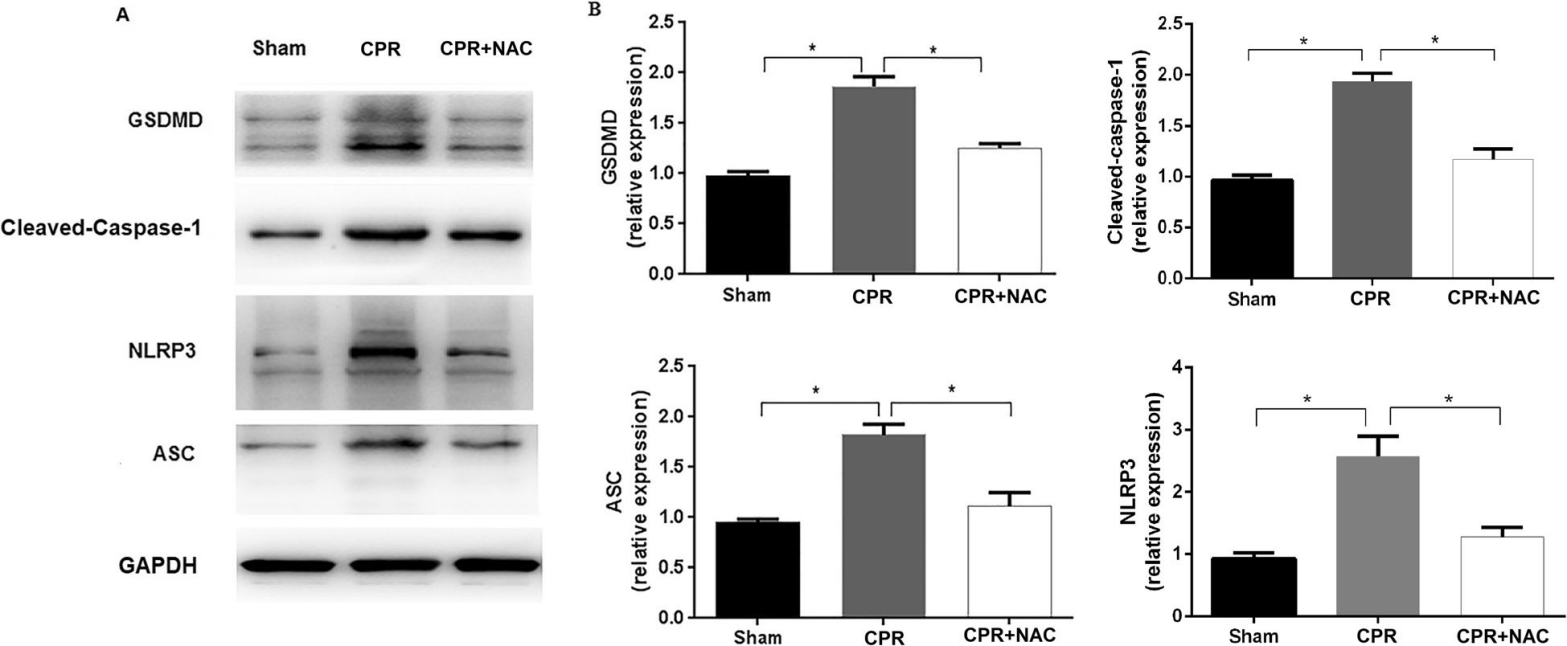
Expressions of NLRP3, ASC, Cleaved-Caspase-1, and GSDMD, and the effect of NAC on myocardial pyroptosis after ROSC. **a** Representative western blotting images of NLRP3, ASC, Cleaved-Caspase-1, and GSDMD proteins in the myocardial tissues at 6 h after ROSC. **b** Quantitative protein analysis of NLRP3, ASC, and Cleaved-Caspase-1, and GSDMD expression in the three groups at 6 h after ROSC. The columns represent means ± SD of three independent experiments. NAC, N- acetylcysteine; CPR group, *n* = 16; CPR + NAC treatment group, *n* = 16; Sham group, *n* = 12. * *p* < .05 vs. Sham group, *p < .05 vs. CPR + NAC group

To validate the role of NLRP3 inflammasome-mediated pyroptosis in global myocardial I/R injury, we established a cardiac arrest model in SD rats. Protein expression levels of NLRP3, ASC, Cleaved-Caspase-1, and NLRP3 downstream protein GSDMD in myocardial tissues were significantly elevated in the CPR group (Fig. 4a, b). These findings supported the involvement of NLRP3 inflammasome-mediated pyroptosis in global myocardial I/R injury. After use of the antioxidant NAC, results showed a significantly reduction expressions of NLRP3, ASC, Cleaved-Caspase-1, and GSDMD when compared with the CPR group (Fig. 4a, b, *P* < 0.05).

## Discussion

In the present study, our results indicated that post-resuscitation myocardial function deteriorated following the release of ROS and inflammatory cytokines, and we further found that the expression of pyroptosis-related proteins, namely NLRP3, ASC, Cleaved-Caspase1, and GSDMD, were significantly increased in the CPR group. However, treatment with NAC improved myocardial dysfunction, prolonged survival duration, and decreased pyroptosis-related proteins’ expression levels. Therefore, we inferred that NAC, possibly via partly inhibiting ROS-mediated NLRP3 inflammasome-induced pyroptosis, alleviated myocardial dysfunction and improved survival outcomes.

Post-resuscitation myocardial dysfunction contributes to early deaths after resuscitation from cardiac arrest. In our study, we observed a significant reduction of CO and EF, and an increase in MPI after ROSC. However, treatment with NAC improved myocardial dysfunction, including increasing EF and CO and decreasing MPI. This result is consistent with the findings of previous studies that NAC effectively ameliorates myocardial dysfunction [23, 24]. Following cardiac arrest and reperfusion, oxidative stress is generated and its production creates an imbalance between oxidants and antioxidants, which contribute to post-resuscitation myocardial dysfunction. Inflammation is another well-known process after myocardial I/R injury, leading to death of cardiomyocytes. In our study, we assessed oxidative stress and inflammatory factors after ROSC, and established that oxidative stress and inflammation were involved in myocardial dysfunction, as other studies have similarly shown [11–13].

Myocardial I/R injury is a pathological process causing the death of cardiomyocytes, especially when coronary perfusion is restored. Previous evidence has highlighted the key role of the NLRP3 inflammasome in mediating adverse inflammatory responses after myocardial I/R injury [25, 26] and inducing pyroptosis [22, 27]. The NLR3 inflammasome complex including NLRP3, ASC and pro-caspase-1, cleaves and activates caspase-1, then caspase-1 cleaves GSDMD, the later initiates pyroptosis and controls the release of proinflammatory cytokines [28, 29]. In previous study, it has been demonstrated that local myocardial I/R injury can induce increased expression of NLRP3 inflammasome in myocardial tissues [30]. In this study, we established a cardiac arrest model and detected NLRP3 inflammasome and GSDMD in myocardial tissues after global myocardial I/R injury. We found that the expressions of NLRP3, ASC, Cleaved-Caspase-1, and GSDMD in myocardial tissues were significantly increased, which was accompanied by elevated levels of inflammatory cytokines after ROSC. This result is consistent with the findings of local myocardial I/R injury [13]. These findings suggest that NLRP3 inflammasome complex is formed and activated in pyroptosis during the process of global myocardial I/R injury. Furthermore, after treatment with NAC, the expressions of these four proteins were markedly decreased when compared to the CPR group. This means that NAC can inhibit NLRP3 inflammasome induced pyroptosis.

A number of endogenous and exogenous crystalline molecules have been shown to activate the NLRP3 inflammasome [31], however, the mechanism remains unknown. Two events that are common to all activators of NLRP3 inflammasome are a potassium efflux and the generation of ROS [6, 32–35]. During the process of I/R injury, reperfusion after ischemia insults to the myocardium, which is always accompanied by exorbitant ROS formation that induces further myocardial damage [36]. ROS formation and oxidative stress have been shown to be important promoters of NLRP3 inflammasome activation [37–39]. In our study, we detected the levels of oxidative stress, including an increase in MDA and a decrease in SOD, treatment with NAC resulted in a decrease in MDA, and an increase in SOD following a significant reduction of the expressions of NLRP3 ASC Cleaved-Caspase-1 and GSDMD. All results confirmed that there was a relationship between ROS and NLRP3 inflammasome-induced pyroptosis, and NAC improved myocardial dysfunction possibly via partly inhibiting ROS-mediated NLRP3 inflammasome induced pyroptosis.

NAC, as a mucolytic agent, has been used clinically for many years. As the only antidote for acetaminophen poisoning, it was approved by the US Food and Drug Administration in 1985, and the safety of the drug has been well established [40]. The main causes of I/R injury are ROS and inflammation, and therefore NAC has the ability to prevent myocardial I/R injury [41, 42]. In our study, we also verified that oxidative stress and inflammation were present in global myocardial I/R injury, and NAC reduced the production of them. Therefore, as a traditional medicine, NAC might also be used as a promising treatment in protection of post-resuscitation myocardial dysfunction.

Several limitations of this study should be noted. First, we only investigated one timepoint and did not conduct pilot experiments to choose the optimal concentration of NAC. However, we have reviewed many studies before determining the point of administration and the concentration, NAC was given after ROSC, and 150 mg/kg was used as the concentration for our study [43]. Second, there was no histopathology examination of rat heart tissue. The pathological change of I/R injury is well studied before [44]. Third, we didn’t delve into the mechanism of NAC, in this study, we focused on the mechanism of the animal model, and we didn’t explore the mechanism at the cellular level to further clarify how NAC specifically interacts with ROS, and how ROS activates the NLRP3 inflammasome. Fourth, we only assessed the effects of NAC from the short term, without evaluating the long-term changes. Finally, in a 72-h timepoint, we only carried out survival research, and didn’t assess myocardial, neurological dysfunction, and longitudinal changes. In fact, we would like to do more analysis at this timepoint in future studies. In the present study, the CPR group animals all died, so there were no comparisons to investigate. Future studies are warranted to clarify these issues.

## Conclusions

In conclusion, NAC improves post-resuscitation myocardial dysfunction and prolongs the duration of survival by partly inhibiting NLRP3 inflammasome-induced pyroptosis in a rat model of cardiac arrest.

## Methods

All animals received humane care in compliance with the “Principles of Laboratory Animal Care” formulated by the National Society for Medical Research and the Guide for the Care and Use of Laboratory Animals published by the National Institutes of Health [45, 46]. The protocol was approved by the Institutional Animal Care and Use Committee of Tang Wanchun Laboratories of Emergency & Critical Care Medicine.

### Animal preparation

A total of 65 healthy male Sprague-Dawley rats (body weight, 450–550 g) were purchased from the Experimental Animal Center of Beijing, China. Animals were fasted overnight except for free access to water. The animals were anesthetized by intraperitoneal injection of pentobarbital (45 mg/kg). Additional pentobarbital (10 mg/kg) was administrated as needed to maintain anesthesia. A 14G cannula (Abbocath-T, North Chicago, IL) was orally intubated in the trachea. Through the left femoral artery, a 23G polyethylene 50 (PE-50) catheter (Abbocath-T, North Chicago, IL) was advanced into the descending aorta for the measurement of mean arterial pressure (MAP). Through the left external jugular vein, another PE-50 catheter was inserted into the right atrium to measure right atrial pressure. A 3F-PE catheter (model C-PMS-301 J, Bloomington, IN) was inserted through the right external jugular vein into the right ventricle. A preserved guide-wire was then advanced through the 3F-PE catheter into the right ventricle to induce ventricular fibrillation (VF). A thermocouple microprobe (9030–12-D-34; Columbus Instruments, Columbus, OH) was inserted into the left femoral vein to monitor blood temperature. The temperature was maintained at 37 ± 0.5 °C with the aid of a cooling blanket or infrared surface heating lamps. End-tidal CO_2_ was continuously monitored by a Capstar-100 CO_2_ analyzer (IITC Life Science, Ardmore, PA). Electrocardiogram of a conventional lead II was continuously recorded. In addition, all catheters were flushed intermittently with saline containing 2.5 IU/mL heparin.

### Experimental procedures

After surgery, the animals were randomly divided into three groups: (1) sham group (*n* = 12), animals underwent the same operation without the induction of cardiac arrest; (2) CPR group (*n* = 24), animals underwent the induction of cardiac arrest and were administered a placebo (0.9% saline) for control at 5 min after ROSC; and (3) CPR + NAC group (*n* = 24), animals underwent the induction of cardiac arrest and received 150 mg/kg NAC by intravenous injection [43, 47] at 5 min after ROSC.

Fifteen minutes after the baseline measurements were obtained, mechanical ventilation was established with a tidal volume of 0.60 mL/100 g and a respiratory rate of 100 beats/min. The Fio_2_ was maintained at 0.21. VF was electrically induced with a gradual increase in a 60-Hz current to a maximum of 3.5 mA, which was delivered to the right ventricular endocardium. The current flow was maintained for 3 min to prevent spontaneous defibrillation. Mechanical ventilation was discontinued after the onset of VF. After 8 min of untreated VF, CPR was initiated and mechanical ventilation was initiated at a frequency of 100 beats/min. Precordial compression (PC) was synchronized with equal compression and relaxation and a compression/ventilation ratio of 2:1 for 8 min. The depth of compression was adjusted to maintain a coronary perfusion pressure (CPP) at 22 ± 2 mmHg. After 5 min of PC, epinephrine (0.01 mg/kg) was administered through the right atrium. Defibrillation was attempted with up to a 4-J counter shock after 8 min of CPR. If ROSC was not achieved, another 30 s of CPR was performed and a sequence of 4-J shocks was delivered. This procedure was repeated for a maximum of 3 cycles. When supraventricular rhythm returned with MAP more than 50 mmHg for 5 min, ROSC was considered achieved. The resuscitated animals in each group were then randomly divided into two subgroups: ROSC 6 h and ROSC 72 h. Rats in the ROSC 6 h subgroup received an echocardiography at baseline and 1, 2, 4, and 6 h after ROSC, and blood drawing at baseline, 1 and 6 h after ROSC under anesthesia. The heart was then rapidly excised and some sections were stored in refrigeration at − 80 °C for further testing. In the ROSC 72 h subgroup, all catheters were removed at 6 h after ROSC. Then, the animals were returned to their cages and closely monitored until 72 h after ROSC.

### Echocardiography

Myocardial function was measured by ultrasound (Model SONIX OP; Ultrasonix Medical Corporation, Richmond, Canada), utilizing a 12.5-Hz transducer at baseline and 1, 2, 4, and 6 h after ROSC. CO and EF were adopted to estimate the myocardial contractility. EF was calculated using the area-length method as described in previous research [48]. MPI, which combines time intervals related to systolic and diastolic function, was calculated according to the formula (a–b)/b, where a = mitral valve closure-to-opening interval and b = left ventricular ejection time. All measurements were taken and confirmed by two separate investigators [49].

### Enzyme-linked Immunosorbent Assay (ELISA)

The levels of IL-1β, IL-6, SOD and MDA in serum at baseline, ROSC 1 h, and ROSC 6 h were measured using an IL-1β rat ELISA kit and IL-6 rat ELISA kit, and a SOD assay kit and MDA assay kit, according to the manufacturers’ instructions.

### Western blot

The homogenized heart tissues were centrifuged at 12,000 rpm for 10 min at 4 °C, and supernatant was obtained. The total protein concentration was measured by standard BCA Protein assay. Proteins (20 μg–40 μg) were loaded to a 10% sodium dodecyl sulfate (SDS) polyacrylamide gel, and after electrophoresis, transferred to polyvinylidene fluoride membranes (PVDF) (Millipore). After then, the PVDF membranes were blocked using 5% defatted milk in Tris-buffered saline with Tween-20 (TBST) for 1 h at room temperature. Then, the membranes were incubated with primary antibodies for GSDMD (1:1000, Rat, Abcam), NLRP3 (1:1000, Rat, Cell Signaling Technology), ASC (1:500, Rat, Santa Cruz), and Cleaved-Caspase 1 (1:1000, Rat, Santa Cruz), overnight at 4 °C. Next, the membranes were washed with TBST three times, and incubated with secondary antibodies for 1 h at room temperature. Finally, the PVDF membranes were washed once more in TBST and analyzed using an enhanced chemiluminescence detection kit (BeyoECL Plus, Beyotime, China). The band intensities were quantified by Image J software (NIH).

### Statistical analysis

Primary study outcomes were continuous and presented as mean ± SD. Normality was checked using Shapiro-Wilk test. One-way repeated-measure analysis of variance (ANOVA) was used to conduct omnibus comparisons on parameters among the three groups. Pairwise differences among the groups were tested post hoc with Tukey’s test and student’s *t* test. The survival rates among the groups were obtained using Kaplan-Meier survival estimates and compared with the log-rank test. Throughout, *p* values of less than 0.05 were considered statistically significant. All statistical analyses were performed using SPSS (version 22.0 for Windows).

## Data Availability

The data sets in the current study are available from the corresponding author on reasonable request.

## Abbreviations

NLRP3: NOD-like receptor 3
ROS: Reactive oxygen species
I/R: Ischemia/reperfusion
NAC: N-acetylcysteine
GSDMD: Gasdermin D
MDA: Malondialdehyde
SOD: Superoxide dismutase
ROSC: Return of spontaneous circulation
CPR: Cardiopulmonary resuscitation
VF: Ventricular fibrillation
